# Preoperative Arterial and Venous Cannulation in Redo Cardiac Surgery: From the Safety and Cost-effectiveness Points of View

**DOI:** 10.21470/1678-9741-2019-0472

**Published:** 2020

**Authors:** Yahya Yildiz, Mustafa Ozer Ulukan, Korhan Erkanli, Orcun Unal, Didem Melis Oztas, Metin Onur Beyaz, Murat Ugurlucan

**Affiliations:** 1 Department of Anesthesiology, Medical Faculty, Istanbul Medipol University, Istanbul, Turkey.; 2 Department of Cardiovascular Surgery, Medical Faculty, Istanbul Medipol University, Istanbul, Turkey.; 3 Cardiovascular Surgery Clinic, Yedikule Chest Diseases and Thoracic Surgery Training and Research Hospital, Istanbul, Turkey.; 4 Cardiovascular Surgery Clinic, Bagcilar Training and Research Hospital, Istanbul, Turkey.

**Keywords:** Cardiopulmonary Bypass, Extracorporeal Membrane Oxygenation, Sternotomy, Catheterization, Cost-Benefit Analysis

## Abstract

**Objective:**

To investigate the safety and cost-effectiveness of preoperative cannulation and conventional approach techniques.

**Methods:**

Sixty-one patients who underwent redo open cardiac procedures between September 2015 and November 2018 were divided into two groups - Group A (n: 30), patients who underwent conventional cannulation after sternotomy, and Group B (n: 31), those who underwent cannulation before sternotomy. Patients were evaluated retrospectively for general complication rates and total hospital costs.

**Results:**

Mortality occurred in four patients from Group A and in one patient from Group B. Four patients required extracorporeal membrane oxygenation (ECMO) in Group A, whereas two required ECMO in Group B. Duration of total operation, cardiopulmonary bypass, and cross-clamp times were longer in the conventional surgery group than in the pre-sternotomy cannulation group (420.29±188.84 *vs.* 314.77±187.38, *P*=0.036; 171.87±85.59 *vs*. 141.7±82.47, *P*=0.089; and 102.94±70.67 *vs*. 60.97±52.81, *P*=0.009; respectively). Total blood and blood product usage were higher in Group A than in Group B. Postoperative intensive care unit stay was 62.77±145.3 hours *vs*. 25.13±73.11 hours, ventilation time was 5.16±5.09 hours *vs*. 3.03±2.78 hours, duration of ward stay was 5.23±2.52 days *vs*. 5.57±2.16 days, and duration of hospital stay was 9.58±5.85 days *vs*. 9.8±5.31 days in conventional sternotomy and pre-sternotomy cannulation groups, respectively. Total hospital costs were calculated 35863.52±20803.99 Turkish Liras (TL) in Group A and 25744.74±16472.03 TL in Group B (*P*=0,042).

**Conclusion:**

Venous and arterial cannulations before sternotomy decreased myocardial injury and complication rates, blood and blood product usage, hospital stay, and, consequently, hospital costs in our modest cohort.

**Table:** 

Abbreviations, acronyms & symbols				
**ASD**	**= Atrial septal defect**	** **	**MVR**	**= Mitral valve replacement**
**AVI**	**= Aortic valve insufficiency**		**ns**	**= Non-significant**
**AVR**	**= Aortic valve replacement**		**Op.**	**= Operation**
**AVS**	**= Aortic valve stenosis**		**PA**	**= Pulmonary artery**
**AVSD**	**= Atrioventricular septal defect**		**PS**	**= Pulmonary stenosis**
**BSA**	**= Body surface area**		**PVI**	**= Pulmonary valve insufficiency**
**CABG**	**= Coronary artery bypass grafting**		**PVR**	**= Pulmonary valve replacement**
**CAD**	**= Coronary artery disease**		**RVOT**	**= Right ventricle outflow tract**
**CC**	**= Cross-clamping**		**TL**	**= Turkish Lira**
**CPB**	**= Cardiopulmonary bypass**		**TOF**	**= Tetralogy of Fallot**
**CT**	**= Computerized tomography**		**TVI**	**= Tricuspid valve insufficiency**
**ECMO**	**= Extracorporeal membrane oxygenation**		**TVP**	**= Tricuspid valvuloplasty**
**LPV**	**= Left pulmonary vein**		**TVR**	**= Tricuspid valve replacement**
**MS**	**= Mitral stenosis**		**USG**	**= Ultrasonography**
**MVI**	**= Mitral valve insufficiency**		**VSD**	**= Ventricular septal defect**

## INTRODUCTION

Cardiovascular surgery procedures are very frequently performed worldwide. The number of patients undergoing open-heart surgery increased as the increase in life expectancy. In addition, the number of patients necessitating redo surgery for primary cardiac disease or other cardiovascular pathologies and requiring resternotomy is not low. Despite advances in medicine, technology, and techniques, redo cardiac surgery is still challenging and carries certain mortality and morbidity rates^[1]^, as well as consequent increased blood and blood product usage, hospitalization requirement, and, therefore, increased hospital costs.

Redo cardiac surgery may be performed with standard aortic and venous cannulations after sternotomy or, alternatively, with arterial and venous cannulations prior to sternotomy. In this manuscript, we aimed to compare the conventional cannulation method with the preoperative cannulation technique regarding complication rates and total hospital costs.

## METHODS

Between September 2015 and December 2018, 61 patients who underwent redo cardiac surgery procedures were retrospectively investigated. The patients were divided into two groups - Group A, 30 patients who were operated with conventional aortic and venous cannulation (two-stage atrial or bicaval, depending on the surgery), and Group B, 31 patients in whom sternotomy was performed after jugular venous and femoral arterial and venous cannulations. Patients who required emergency surgery or reexplorations to assist device insertion were excluded from the study. Patients weighting < 20 kg were not included either into Group A or Group B due to the unavailability of suitable superior vena cava, femoral vein, and femoral artery cannulas in order to overcome bias. All patients underwent doppler ultrasonography (USG) examination of the femoral and neck vessels to measure the vessels’ size and their course to rule out any stenosis or occlusions preoperatively. The adequacy of the vessel’s diameter is determined in accordance with the predetermined cannula size adjusted to the body surface area of each patient.

Demographic features, perioperative findings, operative events, blood and blood product usage, duration of ventilation, intensive care unit and hospital stays, extracorporeal membrane oxygenation (ECMO) usage, mortality and morbidity, and total hospital cost were recorded and compared between the two groups.

### Statistical Analysis

Collected data were analyzed with the IBM SPSS Statistics software, version 20. Descriptive statistics (absolute frequencies and percentages for categorical variables, means and standard deviation for continuous variables) were used to evaluate demographic and clinical characteristics of the population. Values are expressed as mean ± standard deviation or frequency and percentage. For continuous variables, conformity of normal distribution and homogeneity were tested with the Kolmogorov-Smirnov test. Categorical values were evaluated with Chi-squared test and parametric values were evaluated with independent samples t-test. The differences between the two groups for consecutive measurements were evaluated with repeated measures analysis of variance. A P<0.05 was considered statistically significant.

## RESULTS

Among 30 patients who underwent surgical procedures with conventional methods, there were 11 adults and 19 pediatric cases. Nineteen of them were males and 11 were females. There were 13 adults and 18 pediatric patients in Group B, and 21 of the patients were male, whereas remaining 10 were females. Mean age of the patients in Group A was 26,06±25,15 (6-72) years and it was 27,6±22,05 (6-70) years in Group B. Body mass indices were calculated 1,30±0,51 (0.72-2.32) kg/m^2^ and 1,44±0,44 (0.74-2.07) kg/m^2^ in Group A and B, respectively. Age, gender, weight, height, and body mass indices were not significantly different between the two groups (Table 1).

**Table 1:** Demographic data of the patients in the conventional cannulation group and the preoperative USG-guided cannulation group.

	Age (year)	High (cm)	Weight (kg)	Gender (male/female)	Adult/pediatric	BSA (m^2^)
Conventional (n=30)	25.19±25.94	139.61±28.31	45.47±27.10	19-Nov	Nov-19	1.30±0.51
USG (n=31)	27.6±22.05	150.13±23.90	52.35±24.17	21-Oct	13/18	1.44±0.44
*P*-value	0.367	0.53	0.141	ns	ns	0.11

BSA=body surface area; USG=ultrasonography

Diagnosis of the patients who underwent surgery with conventional cannulation techniques are presented on Table 2. Table 3 presents the diagnosis of the patients who were preoperatively cannulated with USG guidance. Eight patients were previously operated two times and three patients were previously operated three times in Group A, whereas seven patients were previously operated two times and three patients were previously operated two times in Group B. All the remaining patients were operated only once and current operations were their second procedures. In Group B, two patients had small caliber superior vena cava and these could not be cannulated; however, since the femoral vein and artery sizes were adequate, these patients underwent operation with femoral arterial and venous cannulations and the superior vena cava was cannulated after median sternotomy.

**Table 2:** Features of the conventional cannulation group (diagnosis, operation type, and durations).

Diagnosis	Op. type	Total CPB (min)	CC (min)	Op. time (min)
Op. ascending aortic dilatation + AVR + AVI	Benthall	116	70	267
AVI + TVI + aortopulmonary window	AVR + TVR + aortopulmonary window	240	129	544
MVI	MVR	116	42	267
VSD + PVI	VSD closure + PVR	84	58	310
AVI + AVS	AVR	114	88	272
MVI + TVI	MVR + TVP	117	58	263
AVI + MVI	AVR + MVR	303	275	744
Op. Jatene+subaortic ridge resection	Subaortic ridge resection	72	38	163
Op. Glenn + left PA stenosis	Fontan + left PA enlargement	204	80	448
ASD + VSD + left PA reconstruction	VSD + ASD + left PA reconstruction	254	164	590
Op. Glenn + main PA reconstruction	Fontan + PA reconstruction	183	0	366
Op. Glenn	Fontan	94	0	300
Op. Glenn + main and left PA reconstruction	Fontan + PA reconstruction	157	122	375
Op. Jatene + ascending aortic dilatation	Op. Jatene+aortic aneurysm repair	189	61	408
AVI + aortic stenosis + MVI + CAD	AVR + MVR + CABG	390	240	900
VSD + main PA reconstruction	VSD + PA plasty	112	73	260
MVI + subaortic ridge + LPV - left atrium connection	MVR + subaortic ridge + LPV left atrium connection	268	224	648
Op. Jatene + Op. VSD + MVI	VSD patch revision + mitral plasty + Op. Jatene	204	142	479
MS + MVI + TVI	MVR + TVP	154	104	360
Op. Benthall	Op. Benthal+CABG	156	37	330
MVI	Mitral cleft repair	74	44	240
MVI + subaortic ridge + main PA stenosis	MS+ subaortic ridge + subpulmonary stenosis repair	268	230	651
Op. Rastelli	Rastelli	218	127	499
Op. TOF	PVR + VSD	58	101	230
VSD + RVOT stenosis	VSD + RVOT reconstruction	144	95	335
Op. Fontan	Fontan	131	106	315
Op. CABG	CABG	370	146	813
MVI + TVI	MVR + TVR	120	46	263
Op. CAD	CABG	110	40	240
Op. Rastelli	Rastelli	270	199	639
Op. Fontan	Fontan	38	52	510

ASD=atrial septal defect; AVI=aortic valve insufficiency; AVR=aortic valve replacement; AVS=aortic valve stenosis; CABG=coronary artery bypass grafting; CAD=coronary artery disease; CC=cross-clamping; CPB=cardiopulmonary bypass; LPV=left pulmonary vein; MS=Mitral stenosis; MVI=mitral valve insufficiency; MVR=mitral valve replacement; Op=operation; PA=pulmonary artery; PVI=pulmonary valve insufficiency; PVR=pulmonary valve replacement; RVOT=right ventricle outflow tract; TOF=tetralogy of Fallot; TVI=tricuspid valve insufficiency; TVP=tricuspid valvuloplasty; TVR=tricuspid valve replacement; VSD=ventricular septal defect

**Table 3:** Features of the preoperative USG-guided cannulation group (diagnosis, operation type, and durations).

Diagnosis	Op. type	Total CPB (min)	CC (min)	Op. time (min)
TVI	TVR	116	0	232
Op. TOF ASD	ASD closure	46	15	148
MVI	MVR	112	63	255
TOF + AVI + MVI + PVI	VSD closure + AVR + MVR + TVP + PVR	456	268	1046
AVI	AVR + Op. David	251	150	577
AVI+MVI+TVI	MVR + TVR	214	101	478
Ascending aortic dilatation	Ascending aortic replacement	106	24	224
MVI + TVI	MVR + TVP	155	67	343
ASD	ASD closure	138	31	291
ASD	ASD closure	90	29	194
Op. Fontan + narrow VSD	Tricuspid valve + ASD enlargement	123	64	278
TVI	TVR	69	0	138
Op. Glenn	Op. Fontan	167	77	372
Subaortic stenosis + AVI+ aortic stenosis	AVR + subaortic ridge + Op. Marlow	109	72	254
AVI + subaortic stenosis	AVR + subaortic ridge repair	89	57	206
MVI + TVI + CAD	MVR + CABG (X1)	168	92	382
MVI + Op. Glenn - anastomosis stenosis	Mitral plasty, Op. Glenn	148	23	307
MVI + TVI + CAD	MVR + TVP + CABG (X2)	135	80	310
Op. Glenn	Op. Fontan	125	63	281
Op. Glenn	Op. Fontan	104	0	208
Pulmonary regurgitation + VSD	PVR + VSD + closure	68	42	136
Op. AVSD	ASD	65	25	142
Op. Glenn + Op. Fontan + subaortic stenosis	Fontan + subaortic stenosis repair + PA reconstruction + VSD enlargement	278	72	592
Op. AVSD	ASD	114	46	251
Op. Glenn	Op. Fontan + subaortic stenosis + PA reconstruction	285	144	642
Op. Fontan + narrow VSD	VSD enlargement + Op. Fontan	79	35	180
MVI	MVR	89	51	203
Op. TOF + catheter/PVR was not successful	ASD + PS	100	60	230
Op. TOF + left PA stenosis + PS+ catheter was performed urgently and it was not successful	Left PA enlargement + PVR	142	18	293
Op. AVR+Op. MVR/pannus	Aortic valvuloplasty + MVR	110	60	250

ASD=atrial septal defect; AVI=aortic valve insufficiency; AVR=aortic valve replacement; AVSD=atrioventricular septal defect; CABG=coronary artery bypass grafting; CAD=coronary artery disease; CC=cross-clamping; CPB=cardiopulmonary bypass; MVI=mitral valve insufficiency; MVR=mitral valve replacement; Op.=operation; PA=pulmonary artery; PS=pulmonary stenosis; PVI=pulmonary valve insufficiency; PVR=pulmonary valve replacement; TOF=tetralogy of Fallot; TVI=tricuspid valve insufficiency; TVP=tricuspid valvuloplasty; TVR=tricuspid valve replacement; VSD=ventricular septal defect

### Operative Events

Measured with USG, and most probably secondary to angiographic interventions, the right femoral artery calibers (three patients) and the right femoral vein calibers (four patients) were too small to deploy an adequate size cannula, and these patients received left femoral arterial and venous cannulations. In one patient, bilateral femoral arterial and venous sizes were small and femoral cannulation could not be performed.

Duration of operation was 420,29±188.84 (163-900) minutes in the conventional surgery group, whereas it was 314.77±187.38 (136-1046) minutes in the USG cannulation group (P=0.0366). Mean cardiopulmonary bypass times were not significantly different between the two groups - Group A, 171,87±85,59 (74-370) minutes vs. Group B, 141.7±82.47 (46-456) minutes; P=0.089. The cross-clamp times differed significantly between the two groups as 102.94±70.67 (0-275) minutes in Group A and 60.97±52.81 (0-268) minutes in Group B (P=0.009).

Operative events included atrial, ventricular, and major arterial injuries and requirement of total circulatory arrest. During sternotomy, cardiac and major vascular injuries occurred in seven patients from the conventional surgery group (22.5%); three of them were atrial injuries, one was ventricular injury, two were innominate vein injuries, and one was aortic injury; whereas in the USG group, there was only one atrial and one right ventricular injuries (in total, two patients or 6,7%) (P=0.048). Total circulatory arrest was required in four patients from Group A in order to repair the major injuries. The mean duration of total circulatory arrest was 11.5±2.05 (13, 11, 8, and 14) minutes. Circulatory arrest was not required in any of the cases from Group B. Femoral artery injury occurred in five patients from Group A and in one patient from Group B (P=0,092). Acidosis secondary to blood loss occurred at the pre-bypass stage and it was observed in four and two patients in conventional and USG groups, respectively (P=0.212). Blood loss was replaced with erythrocyte suspension to achieve hemoglobin levels > 9 mg/dl.

### Postoperative Findings

Mortality occurred in four patients from the conventional cannulation group and in one patient from the USG group (P=0.092). Three of them died from multiorgan failure, one from congestive heart failure, and one from sepsis. Mortalities were observed on the second, 16^th^, 21^st^, and 25^th^ postoperative days in Group A. The last patient could be discharged from the intensive care unit to the ward; however, he needed to be taken back to the intensive care unit after three days and put on ECMO due to low cardiac output heart failure. He was lost after four days. In the USG group, the only mortality occurred on the 17^th^ postoperative day, although the patient was on ECMO therapy. Major infections occurred in three patients from Group A (mediastinitis, sternal osteomyelitis, and pneumonia) and one patient from Group B (sternal deep wound infection); overall, four patients in both groups (P=0.08).

Extracorporeal membrane oxygenator was used in four cases from Group A and in two cases from Group B (P=0.33). Additional cardiopulmonary bypass was required in five patients from Group A and three patients from Group B (P=0.13) due to difficulties of disconnection from cardiopulmonary bypass. Duration of ventilation did not differ between the two groups, 62.77±145.3 (4-570) hours in the conventional group vs. 25.13±73.11 (4-408) hours in the USG group (P=0.117). Intensive care unit stay differed significantly between groups; it was 5.16±5.09 (2-21) days in Group A and 3.03±2.78 (2-17) days in Group B (P=0,028). Hospital stay was 9,58±5.85 (7-29) days in the conventional group and 9.8±5.31 (6-29) days in the USG group (P=0.33).

When examining the amount of blood and blood products usage, as well as additional thrombotic agents used in the patients, erythrocyte - 5,29±7,26 (2-31) units in Group A and 2.43±2.15 (1-12) units in Group B - and thrombocyte suspension - 2.23±2.95 (1-9) in the conventional cannulation group and 1.06±1.87 (0-8) units in the USG group - usage differed significantly between the groups (P=0.027 and P=0.029, respectively). The usage of fresh frozen plasma - 3.13±1.97 (1-9) units vs. 2.93±5.17 (0-26) units -, cryoprecipitate - 6.32±5.99 (0-16) units vs. 5.06±5.25 units (0-16) -, and Cofact - factors II, VII, IX, and X/NovoSeven (eptacog alfa) (three vs. one and one in each) - did not differ significantly between the groups.

The total hospital cost differed significantly between the groups. Mean hospital cost was calculated 35.863,52±20.803,99 (14.967-109.886) Turkish Liras (TL) in the conventional group, whereas 25,744.74±16,472.03 (15.188-103.666) TL in the USG group (P=0.044) (1 TL is equivalent to 6.03 EURO/5.3 USD). Blood and blood product costs were separately recorded and calculated. Mean cost of blood and blood products was 2.591,21±2.744,42 (573-11.586) TL in the conventional group, which was significantly higher when compared with the USG group, 1,294.35±1,553.29 (217-7.379) TL (P=0.013).

## DİSCUSSİON

The number of patients requiring redo cardiac procedures increased drastically in cardiovascular surgery. These additional procedures may be performed for the correction of the primary procedure or treatment of additional cardiac disorders. The reoperations carry certain risks during reentry. Myocardial or conduit injury, excessive bleeding, increased blood and blood product usage, and inadequate myocardial protection may occur. Most of the events occur during sternotomy and exploration. Technical challenges are due to loss of anatomical planes secondary to adhesions resulting from scarring of tissues after the first operation. In addition, these procedures become even more complex when the patients have additional comorbidities. Despite preventive measures to overcome adhesions and protect the original anatomy, none of the methods (e.g., pericardial closure, usage of adhesion barriers, etc.) have been sufficient to prevent mediastinal scarring^[2]^. There is certain mortality risk during adult and pediatric redo operations reported in the literature^[3]^. Hence, it is of utmost importance to design safe strategies for redo cases.

In addition to preventive measures, preoperative surgical planning and strategy determination are important during redo cases. Chest X-ray examination is a routine component of anesthesia and cardiac procedures. Although it gives clues about the distance between the sternum and mediastinal structures, it is not possible to discriminate the adherent structures. Roentgenogram, echocardiography, or conventional angiography may only be suggestive. Despite the radiation and contrast hazards, computerized tomography (CT) scan has become an increasingly used diagnostic tool for cardiac surgery. Detailed three-dimensional anatomy of the heart and adjacent structures may be obtained with CT examination. Accordingly, the risk of myocardial or conduit injury as well as preventive measures can be precluded by CT of the chest^[2]^. Moreover, CT enables printing three-dimensional images of the heart and chest and makes it possible to practice preoperatively on the same cardiac pathology ex vivo, for a better surgical outcome when needed^[4]^. Hamid et al.^[2]^ proposed mandatory use of CT examination preoperatively to precisely determine the intrathoracic structures, plan a strategy to advance into the mediastinum, decrease the risk of cardiac injury, even without contrast in patients with critical nephrologic status. In general, an enlarged or calcified conduit behind the sternum and absent or insufficient retrosternal space are the major risk factors for sternal reentry^[5]^. The right ventricle, ascending aorta, coronary bypass grafts, and right ventricular outflow tract conduits may adhere to the back table of the sternum after the previous operations. Oscillating saw decreases the risk of myocardial injury; however, it does not eliminate the risks completely. The risks are not over even after mediastinal entry, and cardiac manipulations may lead to arrythmias^[6]^. Catastrophic complications, mainly severe hemorrhage, have been presented in the literature^[7]^.

In contrast, the literature includes various reports indicating that resternotomy could be performed with low risks and acceptable results^[2,7-9]^, and even very brave reports indicating zero moderate or major cardiac injury/catastrophic hemorrhage at reoperation^[10]^. However, safety precautions and extreme care should be taken for an uneventful redo procedure. In our study, we sought to investigate the safety and cost-effectiveness of preoperative ultrasound-guided peripheral cannulation technique and compared it with conventional approach in redo cardiac surgery.

Ultrasound-guided peripheral cannulation for cardiopulmonary bypass may be performed in case of hemodynamic instability, emergency cardiopulmonary bypass requirement, in cases where central cannulation would be impossible or dangerous (such as porcelain aorta or type A aortic dissection), for minimal invasive surgery, or when the likelihood of myocardial or conduit injury precluded before surgery would be high^[2]^. It is some institutions’ policy to routinely cannulate the patients prior to sternotomy to empty the heart and prevent myocardial injury^[6,11,12]^. We randomly divided 61 consecutive patients who were scheduled for redo cardiac surgery at our institution into two groups (conventional sternotomy group and preoperative cannulation group) and investigated the safety and cost-effectiveness of both methods.

The institution of cardiopulmonary bypass before resternotomy may be indicated when there is close proximity of the ascending aorta, saphenous vein, internal thoracic artery grafts, the right ventricle, or in cases of severe tricuspid regurgitation, previous mediastinitis, and in patients presenting with cardiogenic shock and severely compromised ventricular functions, such as an ejection fraction < 25%^[6]^. Preoperative doppler USG examination of the arteries and veins is crucial in order to precisely determine the cannulation strategy; i.e., through femoral vessels, conventionally, or with axillary approach; otherwise, there are serious injuries reported in the literature in patients who did not receive preoperative evaluation^[6]^. Following general anesthesia, the anesthesiology team inserted a jugular venous cannula with doppler USG guidance in our cohort. The diameters of bilateral femoral arteries and veins were measured with doppler USG and appropriate size cannulas were chosen. Open technique was preferred for femoral cannulations. The positions of the cannula were confirmed with transesophageal echocardiography. In our cohort, measurement of the femoral vessels indicated unsuitable bilateral femoral vessels in one patient in the doppler USG group. In the conventional surgery group, femoral regions were draped and left uncovered in case of a severe myocardial injury and cardiopulmonary bypass requirement. Emergency cardiopulmonary bypass with groin incision after major bleeding was required in four patients from the conventional surgery group.

In our cohort, the conventional surgical strategy yielded more perioperative events, as well as increased mortality and morbidity, number of extracorporeal membrane oxygenator usage, duration of mechanical ventilation, blood and blood product usage, intensive care unit and hospital stays, and overall hospital costs when compared with the pre-sternotomy cannulation group. Since the literature lacks studies comparing both techniques in means of hospital costs, we failed to discuss our results considering the healthcare expenses in redo cardiac surgery cases.

### Limitations

The major limitation of the study is the relatively small number of the cohort divided in both groups. The retrospective nature of the research may be regarded as another limitation. CT examinations of the thorax could have been performed in the preoperative period in order to determine the adherent structures and plan the strategy to enter the mediastinal cavity. This may be accounted among the limitations; however, it would bias the study. Since the literature is unavailable for hospital costs of the particular patient groups, i.e., none of the papers in the literature either stating the safety of pre-sternotomy cannulation or stating no difference between conventional sternotomy and prior cannulation groups studied on the healthcare expenses, we were unable to discuss our findings.

## CONCLUSİON

Installation of cardiopulmonary bypass before sternotomy decreased complications, morbidity, and mortality in our modest cohort. The hospital costs were researched in this study, differently than other similar reports in the literature, and we found that pre-sternotomy cannulation is associated with decreased healthcare budget.

**Table:** 

Authors' roles & responsibilities	
YY	Substantial contributions to the conception or design of the work; or the acquisition, analysis, or interpretation of data for the work; drafting the work or revising it critically for important intellectual content; agreement to be accountable for all aspects of the work in ensuring that questions related to the accuracy or integrity of any part of the work are appropriately investigated and resolved; final approval of the version to be published
MOU	Substantial contributions to the conception or design of the work; or the acquisition, analysis, or interpretation of data for the work; drafting the work or revising it critically for important intellectual content; agreement to be accountable for all aspects of the work in ensuring that questions related to the accuracy or integrity of any part of the work are appropriately investigated and resolved; final approval of the version to be published
KE	Substantial contributions to the conception or design of the work; or the acquisition, analysis, or interpretation of data for the work; drafting the work or revising it critically for important intellectual content; agreement to be accountable for all aspects of the work in ensuring that questions related to the accuracy or integrity of any part of the work are appropriately investigated and resolved; final approval of the version to be published
OU	Substantial contributions to the conception or design of the work; or the acquisition, analysis, or interpretation of data for the work; drafting the work or revising it critically for important intellectual content; agreement to be accountable for all aspects of the work in ensuring that questions related to the accuracy or integrity of any part of the work are appropriately investigated and resolved; final approval of the version to be published
DMO	Substantial contributions to the conception or design of the work; or the acquisition, analysis, or interpretation of data for the work; drafting the work or revising it critically for important intellectual content; agreement to be accountable for all aspects of the work in ensuring that questions related to the accuracy or integrity of any part of the work are appropriately investigated and resolved; final approval of the version to be published
MOB	Substantial contributions to the conception or design of the work; or the acquisition, analysis, or interpretation of data for the work; drafting the work or revising it critically for important intellectual content; agreement to be accountable for all aspects of the work in ensuring that questions related to the accuracy or integrity of any part of the work are appropriately investigated and resolved; final approval of the version to be published
MU	Substantial contributions to the conception or design of the work; or the acquisition, analysis, or interpretation of data for the work; drafting the work or revising it critically for important intellectual content; agreement to be accountable for all aspects of the work in ensuring that questions related to the accuracy or integrity of any part of the work are appropriately investigated and resolved; final approval of the version to be published
